# Transcriptomic response of female adult moths to host and non-host plants in two closely related species

**DOI:** 10.1186/s12862-018-1257-3

**Published:** 2018-09-20

**Authors:** M. Orsucci, P. Audiot, S. Nidelet, F. Dorkeld, A. Pommier, M. Vabre, D. Severac, M. Rohmer, B. Gschloessl, R. Streiff

**Affiliations:** 1 CBGP, INRA, CIRAD, IRD, Montpellier SupAgro, Univ Montpellier, Montpellier, France; 2 DGIMI, INRA, Univ Montpellier, Montpellier, France; 3 DIASCOPE, INRA, Mauguio, France; 4 Present address: Department of Ecology and Genetics, EBC, Uppsala University, Norbyvägen 18D, 75236 Uppsala, Sweden; 5 MGX-Montpellier GenomiX, c/o Institut de Génomique Fonctionnelle, 34094 Montpellier Cedex 5, France

**Keywords:** Specialization, Oviposition, Insect-plant interaction, Adaptation, Transcriptomics

## Abstract

**Background:**

Divergent selection has been shown to promote speciation in many taxa and especially in phytophagous insects. In the *Ostrinia* species complex, the European corn borer (ECB) and adzuki bean borer (ABB) are two sibling species specialized to different host plants. The first is a well-known maize pest, whereas the second is a polyphagous species associated with various dicotyledons. Their specialization to host plants is driven by morphological, behavioral and physiological adaptations. In particular, previous studies have shown that ECB and ABB display marked behavior with regard to plant choice during oviposition, involving specific preference and avoidance mechanisms. In this study, our goal was to identify the mechanisms underlying this host-plant specialization in adult females through an analysis of their gene expression. We assembled and annotated a de novo reference transcriptome and measured differences in gene expression between ECB and ABB females, and between environments. We related differentially expressed genes to host preference behavior, and highlighted the functional categories involved. We also conducted a specific analysis of chemosensory genes, which are considered to be good candidates for host recognition before oviposition.

**Results:**

We recorded more differentially expressed genes in ECB than in ABB samples, and noticed that the majority of genes potentially involved in the host preference were different between the two species. At the functional level, the response to plant environment in adult females involved many processes, including the metabolism of carbohydrates, lipids, proteins, and amino acids; detoxification mechanisms and immunity; and the chemosensory repertoire (as expected). Until now, most of the olfactory receptors described in *Ostrinia* spp. had been tested for their putative role in pheromone recognition by males. Here we observed that one specific olfactory receptor was clearly associated with ECB’s discrimination between maize and mugwort conditions, highlighting a potential new candidate involved in plant odor discrimination in adult females.

**Conclusions:**

Our results are a first step toward the identification of candidate genes and functions involved in chemosensory processes, carbohydrate metabolism, and virus and retrovirus dynamics. These candidates provide new avenues for research into understanding the role of divergent selection between different environments in species diversification.

**Electronic supplementary material:**

The online version of this article (10.1186/s12862-018-1257-3) contains supplementary material, which is available to authorized users.

## Background

Speciation processes, by which populations diverge and become reproductively isolated, may be driven by divergent selection between different environments that affect various life history traits directly or indirectly related to fitness [12, 20, 22, 57, 72, 73]. In phytophagous insects, host races are relevant examples of population divergence driven or reinforced by host-plant specialization which may, in some cases, be a key component of reproductive isolation [14, 17, 21, 51]. Host plants directly affect the fitness of herbivores [2] due to their role in nutrition [14, 75], protection [26, 36], and reproduction [2, 83]. Indeed, plant metabolites are known to affect the rate of partner encounter and mating success [15, 62], the level of egg laying [18, 41], the choice of oviposition site of gravid females [67], as well as the development of eggs and larvae [56]. In addition to intrinsic host plant qualities, higher trophic level interactions such as plant-associated microorganisms and parasitoids may also impact realized fecundity by impairing larval performance [3]. The herbivorous host range results from these complex pluritrophic interactions, which occur throughout the entire life cycle of the insects [56].

In holometabolous species such as Lepidoptera, oviposition choice is crucial because, before the imago stage, individuals often have limited mobility and thus depend on a judicious choice of plant by the adult female [67]. The preference-performance hypothesis (or “mother-knows-best” hypothesis) suggests that the host preference of adult females should maximize the fitness of their offspring by ovipositing on the most suitable hosts [30]. In their literature survey, Gripenberg et al. [25] suggested that empirical data did support this hypothesis for a majority of the study cases. Molecular and functional studies highlighted the importance of chemosensory mechanisms for the recognition by ovipositing females of olfactory cues emitted by the plant during egg laying [10, 46]. Interestingly, Trona et al. [80] stressed that, in nature, sex pheromones and plant odors are perceived and coded as a whole, and suggested a dual role of plant signals in habitat selection and premating sexual communication.

European corn borer (ECB, *Ostrinia nubilalis* Hübner) and adzuki bean borer (ABB, *Ostrinia scapulalis* Walker), as defined by Frolov et al. [19], are two sibling species of phytophagous moths, co-occurring in a large part of the European continent. ABB occurs in the Palearctic region from Japan to Western Europe. ECB, which is thought to originate from Western Europe, occurs in Northern Africa, Western Europe, and Western Asia and was introduced in the Nearctic region (Northern America) at the end of the nineteenth century. To date, there is no simple phenotypic or molecular characteristics to distinguish these two species. A recent revision of the genus [19], which compiled dozens of years of genetic and phenotypic data (morphology, pheromones, calling time and molecular markers), has concluded that the best criterion for discriminating between ECB and ABB regarding their reproductive isolation was the type of host plant. Indeed, these two species are specialized to different host ranges: ECB is mainly observed on an agricultural monocotyledon, the maize *Zea mays* L., and ABB on several dicotyledons like mugwort (*Artemisia vulgaris* L.), hop (*Humulus lupulus* L.) and hemp (*Cannabis sativa* L.; [19]). In ECB and ABB, the preference-performance hypothesis has typically been observed through various sampling and independent studies [8, 49, 59], but both species exhibit contrasting behaviors and strategies. In a previous experiment, ECB and ABB adult moths were exposed to three plant environments during their mating and egg laying phases: a pure maize environment, a pure mugwort environment and a mixed environment with maize and mugwort plants [59]. The authors observed a strong preference for maize in ECB gravid females when they had the choice between maize and mugwort, coupled with a significant avoidance of mugwort when this plant was the only possible choice (see Additional file 1: Box S1 summary for details). In contrast, ABB showed a moderate preference for mugwort over maize during oviposition and no avoidance behavior. In addition to its oviposition behavior, higher survival and egg-laying rates were observed in ECB when maize was in its environment, with a preference for maize plants also as a resting site. In ABB, these latter traits were not affected by the plant environment (see Additional file 1: Box S1–2). These results suggested attractive and/or repellent molecules impacting oviposition choice [42] as well as synergetic effects on mating and egg laying success [34]. The olfactory responses of ECB females to plant odors have been previously characterized when they were presented with: *i)* injured and uninjured maize plants [74], *ii)* blends of maize volatiles in specific ratios [55, 78], and *iii)* maize vs. mugwort or hop odors [42]. However, the molecular mechanisms (e.g. genes) behind plant recognition and choice are still unknown.

We present here a comprehensive gene expression analysis of ECB and ABB adult females by means of high throughput RNA sequencing of females placed in various plant environments (sampled from the experiment in [59], see Additional file 1: Box S1–1 for details). We assembled and annotated a de novo reference transcriptome and measured differences in gene expression, first between ECB and ABB, then within each moth species between females confronted to contrasting plant environments: pure maize, pure mugwort, and a mixture of maize and mugwort. Furthermore, we linked differentially expressed (DE) genes to the host preference in ECB and ABB and investigated the functional categories involved. Alongside this exhaustive de novo transcriptomic survey, and because of the importance of chemosensory genes, which are considered as good candidates for host recognition before oviposition, we conducted a specific analysis of a chemosensory gene subset previously characterized in ECB, ABB and other related *Ostrinia* species. As for the de novo transcripts, we measured gene expression differences in these chemosensory genes between ECB and ABB, and within each moth species, between contrasting plant environments. The discovery of candidate molecular mechanisms, combined with our knowledge of specialization patterns, offers an opportunity to better understand the role of the host plants in the divergence between ABB and ECB.

## Results

### ECB- and ABB-ref assemblies and annotation

We sequenced RNA from adult ECB and ABB moths sampled from a previous experiment [59], where phenotypic and behavioral traits in moths faced with different plant environments were measured. Three plant environments were tested: *i)* “maize” conditions where only maize plants were available, they being the usual host for ECB; *ii)* “mugwort” conditions where only mugwort plants were available, they being the usual host for ABB; and *iii)* “choice” conditions where both plants were available (the entire experimental framework is given in Additional file 1: Box S1–1.) Twenty-four RNA samples corresponding to two repetitions, three experimental conditions, two tissues (head-thorax: HT, and abdomen: ABDO) and two moth species (ABB and ECB) were sequenced with HiSeq technology, and raw reads were assembled to build de novo transcripts. Details on raw reads and contaminants per library are given in Additional file 2: Table S1, and the main features of the de novo assemblies (HiSeq only or HiSeq-454 combined) are detailed in Table 1. We observed that combining published 454 transcripts and de novo HiSeq transcripts doubled the length of transcripts (Table 1) compared to de novo HiSeq assembly alone. Taking the longest sequence as the only representative for each unigene resulted in 9415 ECB genes and 9992 ABB genes (Table 1). These sets of genes are hereafter referred as ECB-ref and ABB-ref respectively. Homology searches in genomic and protein databases yielded significant hits for 61% of the ECB-ref genes, of which 80% had an associated GO (Gene Ontology) term. More than 97% of the annotated genes were homologous to Lepidoptera genes, followed by homologs to other insect orders (Additional file 2: Table S2). To a much lesser extent, hits with genes of plants, viruses and bacteria were observed. Among these microorganisms, viruses were most prevalent. Details about their detection and annotations are given in Additional file 2: Table S5-A.

**Table 1:** Sequencing data and principal features of ECB and ABB assemblies

			ECB	ABB
Number of raw reads	HiSeq	after sequencing	632,618,343	655,684,618
		after filtering	466,145,250	498,030,304
Number of unigenes	HiSeq		41,155	42,913
Number of transcripts	HiSeq		41,212	42,969
	HiSeq-454	all transcripts	11,456	12,024
	HiSeq-454	longest transcript per unigene	9415	9992
Transcript N50 length (bp)	HiSeq		530.41	544.16
	HiSeq-454		1221	1260
Assembled transcriptome (Mbp)	HiSeq-454		13.7	15.2

### DE genes in ECB- and ABB-ref: Global pattern

DE gene detection was done in both ECB-ref and ABB-ref by mapping sequencing reads for each experimental condition on de novo transcripts. Qualitative trends were observed via multivariate analyses, and quantitative statistics (e.g. log_2_ fold change, Log2FC hereafter) and models were designed with the “EdgeR” function [70]. Generalized linear models (GLMs) were in particular designed to test for the effects of the moth species (ABB vs. ECB) and experimental conditions (maize, mugwort and choice) on gene expression.

Quantitative and qualitative trends were highly correlated between ECB-ref and ABB-ref analyses (data not shown). Thus for simplicity we will present the results for ECB-ref only. Rather than constructing a mixed reference by pooling ECB and ABB reads, we chose to conduct two analyses to avoid chimeric transcripts between species. On a global analytic scale, we did not observe noticeable differences in the number or nature (function and GO term) of the DE genes. Nonetheless, we specifically searched for ABB-ref genes strongly mapped by ABB reads but not by ECB reads. These genes, which probably did not appear in the ECB-ref, may account for some ABB peculiarities like less stringent host choice. Genes with such patterns were very rare and, to date, they have not hit with a known protein or function (Additional file 2: Table S3).

The overall percentage of mapped reads on the ECB-ref transcripts varied from 64 to 71% in the 12 HT RNA libraries, and from 58 to 65% in the 12 ABDO libraries (Additional file 2: Table S4). The proportion of multiple mapped reads was less than 1% in HT (0.51%) and ABDO (0.39%) libraries. After filtering genes with coverage of 1X or less, we identified a total of 9326 and 9186 genes expressed across all experimental conditions in the HT and ABDO samples, respectively. Most of them (around 98% in HT and 95% in ABDO) were shared across all experimental conditions (Additional file 3: Figure S1). We detected 24 and 17 genes found to be expressed only in ECB HT and ABDO samples, respectively, and 19 and 118 genes found to be expressed only in ABB HT and ABDO samples, respectively. Fisher’s exact test did not reveal any significant enrichment in GO categories for these lists of genes specific to the ECB and ABB libraries.

### DE genes and source of variation: Species and experimental effects

**Fig. 1:**
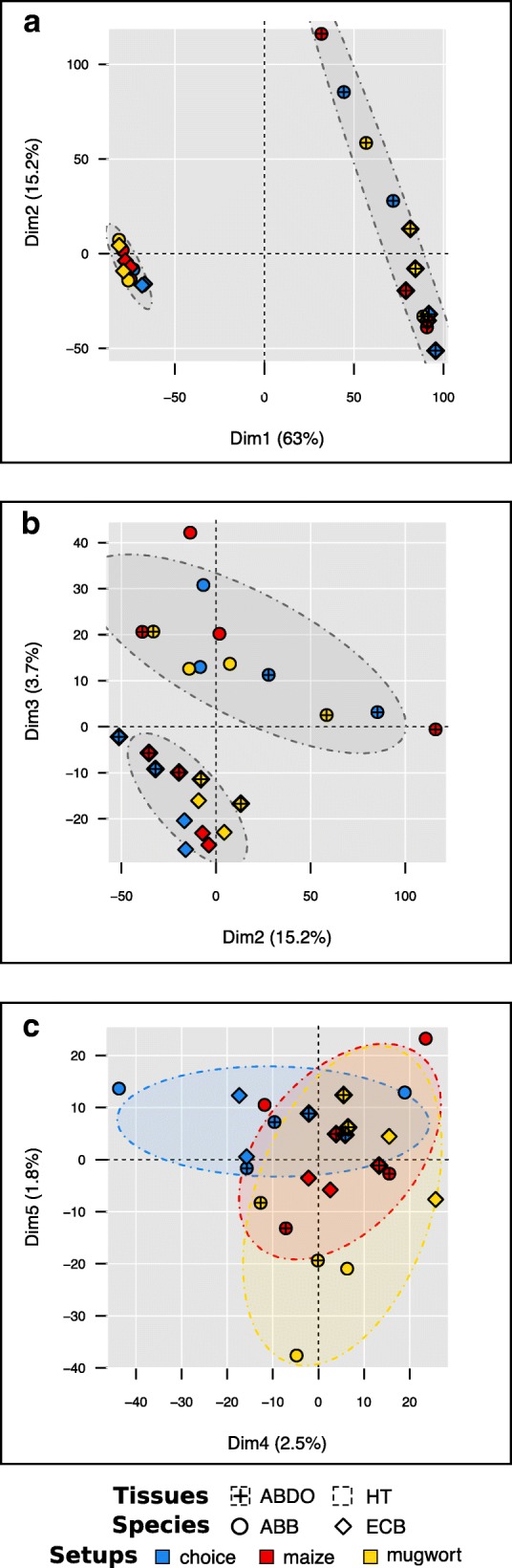
PCA individual factor map of mean raw counts of 9413 genes in 24 RNA libraries. **a** plot on 1–2 axes, ellipses group the libraries’ mean raw counts per tissue (ABDO vs. HT); **b** plot on 2–3 axes, ellipses group the libraries’ mean raw counts per moth species (ECB vs. ABB); **c** plot on 4–5 axes, ellipses group the libraries’ mean raw counts per experimental condition (choice vs. maize vs. mugwort). Species are represented in each plot: ABB (circle) and ECB (diamond). Different colors were used to distinguish between the samples according to the experimental conditions: choice (blue), maize (red) and mugwort (yellow). The percentage of explained variation is indicated on each dimension axis

Apart from the tissues (Fig. 1a), the main source of differential expression was due to contrasts between ECB and ABB samples (Fig. 1b). In HT samples, we detected 337 and 268 transcripts significantly over- and under-expressed in ECB vs. ABB. In ABDO samples, 828 and 132 transcripts were significantly over- and under-expressed in ECB vs. ABB (Table 2, model 2).

**Table 2:** GLM model 1

Models	# libraries	Comparison between	Contrast used (1; − 1)	# DE transcripts	
				HT tissue	ABDO tissue
Model 1: *ti* = modalities^a^	12	moth species	ECB vs. ABB	total = 605	total = 960
				under-expressed = 268	under-expressed = 132
				over-expressed = 337	over-expressed = 828
		experimental condition	Choice vs. Maize	total = 122	total = 9
				under-expressed = 66	under-expressed = 4
				over-expressed = 56	over-expressed = 5
			Choice vs. Mugwort	total = 187	total = 25
				under-expressed = 118	under-expressed = 1
				over-expressed = 69	over-expressed = 24
			Maize vs. Mugwort	total = 129	total = 35
				under-expressed = 86	under-expressed = 1
				over-expressed = 43	over-expressed = 34

^a^“modalities” is a fusion of two variables: moth species with experimental condition

Annotated DE genes in HT tissues between ECB and ABB (Additional file 2: Table S6) were mostly enriched in GO terms (Additional file 2: Table S7) relating to: *i)* DNA/RNA metabolic processes (e.g. reverse transcriptases, transposases, pol proteins and transferases) and *ii)* organic compound metabolic processes (e.g. fructofuranosidases and UDP-glucuronosyltransferases). In most DE genes (|Log2FC| > 2), we also observed several cytochrome P450s involved in detoxification processes. One had previously been characterized in *O. furnacalis* (gb|ACF17813.2, Additional file 2: Table S6) to the DIMBOA toxic compound of maize, and another was an immune-induced protein previously characterized in *Ostrinia* species (gb|AGV28583.1, Additional file 2: Table S6). In ABDO tissues, we observed enrichment in GO terms relating to DNA metabolic processes and molecular binding and transport (Additional file 2: Table S7). In most DE genes (|Log2FC| > 5, Additional file 2: Table S6), we identified apolipophorins potentially involved in immune response [87] as well as the DIMBOA-induced cytochrome P450 previously described in *O. furnacalis* (gb|ACF17813.2, |log2FC| > 5, Additional file 2: Table S6; [88]).

**Fig. 2:**
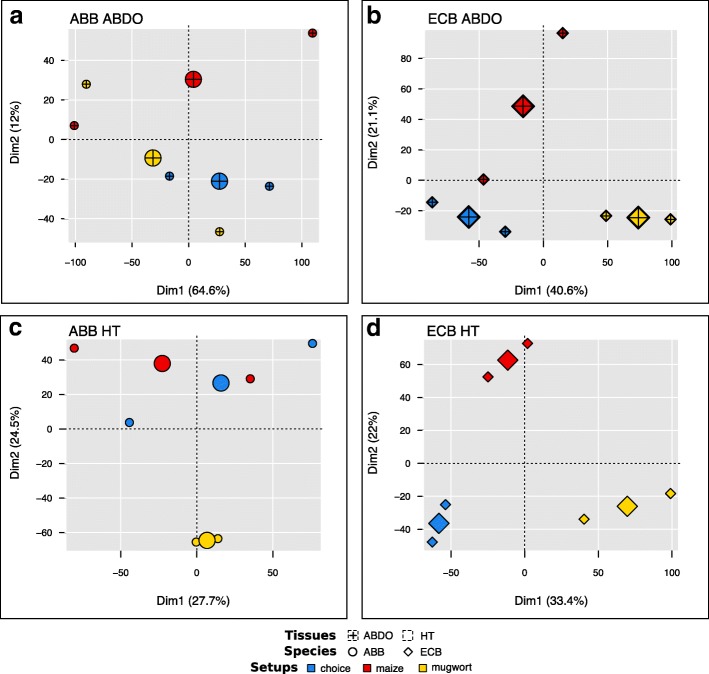
PCA individual factor map of mean raw counts of 9413 genes in six RNA libraries. **a** ABB ABDO samples; **b** ECB ABDO samples; **c** ABB HT samples; **d** ECB HT samples. The small symbol represents each library for each species: ABB (circle) and ECB (diamond). The large symbol represents the barycenter of the related points. Different colors were used to distinguish between the libraries according to the experimental conditions: choice (blue), maize (red) and mugwort (yellow). The percentage of explained variation is indicated on each dimension axis

**Fig. 3:**
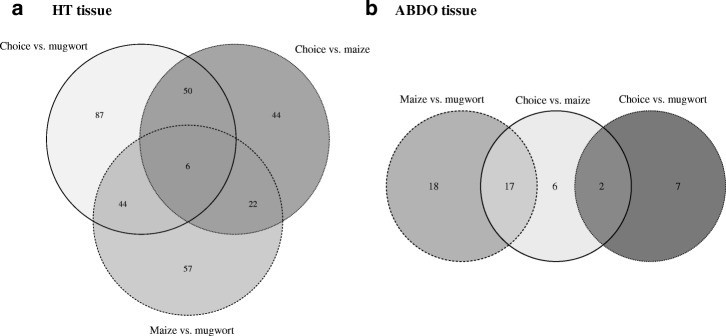
Venn diagram of DE genes between experimental conditions (mugwort vs. choice, maize vs. choice, and mugwort vs. maize) in HT (**a**) and ABDO (**b**) tissues

The experimental condition had the least influence on the variation in gene expression (Table 2, model 2, Fig. 1c). Between 122 and 187 DE genes were observed through comparisons between the experimental conditions (choice vs. mugwort, choice vs. maize, and mugwort vs. maize) in HT samples, and even fewer were observed in ABDO samples (between 9 and 35). These contrasting numbers of DE genes reflected a higher between-repetition dispersion and between-experimental condition overlap in ECB ABDO tissues compared to ECB HT tissues (Fig. 2a-b), as well as in ABB samples vs. ECB samples (Fig. 2c-d vs. Fig. 2a-b). What is more, DE gene lists between the experimental conditions (mugwort, maize and choice) mostly differed from each other (Fig. 3).

Annotated DE genes between experimental conditions (Additional file 2: Table S8) were enriched in a wide variety of GO categories for biological processes (Additional file 2: Table S7) involving feeding and mating behavior, sensing, molecule transport and binding, organic compounds and DNA/RNA metabolism. In most DE genes (|Log2FC| > 5, Additional file 2: Table S8) of the HT and ABDO samples, we identified various enzymes potentially involved in digestion, detoxification and immunity (e.g. glucose dehydrogenase, valine-rich midgut proteins [VMP], heat shock proteins, and cytochrome P450) as well as one protein potentially involved in sensing (transient receptor potential protein).

### DE genes and behavioral traits

**Fig. 4:**
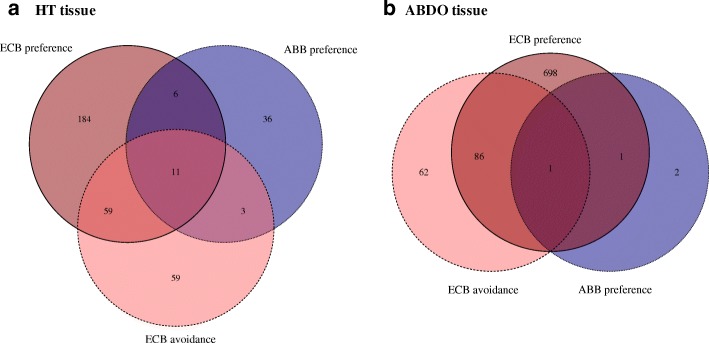
Venn diagram of DE genes involved in ECB-pref, ECB-avoid and ABB-pref in HT (**a**) and ABDO (**b**) tissues. The ECB samples are represented by warm colors and the ABB samples by cool colors

We then focused on DE genes potentially related to specific traits and behaviors observed at the phenotypic level (Additional file 1: Box S1–2), using a specific contrast analysis in the GLM procedure of the EdgeR function (Table 3). We categorized DE genes identified by model 3 (Table 3) for maize preference in ECB (ECB-pref), mugwort avoidance in ECB (ECB-avoid) and mugwort preference in ABB (ABB-pref). Remarkably, many more DE genes were observed in ECB (260 and 786 for ECB-pref, and 132 and 149 for ECB-avoid; Tables 3 and 4, and more details in Additional file 2: Table S9) than in ABB (56 and 4 for ABB-pref, Tables 3 and 4, and more details in Additional file 2: Table S9). Moreover, the preference and avoidance gene lists mostly differed from each other (Fig. 4 and Additional file 2: Table S10).

**Table 3:** GLM model 2

Targeted trait	# libraries	Models	Contrast used (0.5; 0.5; −1)	# DE transcripts	
				HT tissue	ABDO tissue
Maize preference in ECB	6	Model 3: *ti* = experimental condition	Choice and Maize vs. Mugwort	total = 260	total = 786
				under-expressed = 148	under-expressed = 608
				over-expressed = 118	over-expressed = 178
Mugwort avoidance in ECB			Choice and Mugwort vs. Maize	total = 132	total = 149
				under-expressed = 84	under-expressed = 128
				over-expressed = 48	over-expressed = 21
Mugwort preference in ABB	6		Choice and Mugwort vs. Maize	total = 56	total = 4
				under-expressed = 15	under-expressed = 3
				over-expressed = 41	over-expressed = 1

**Table 4:**
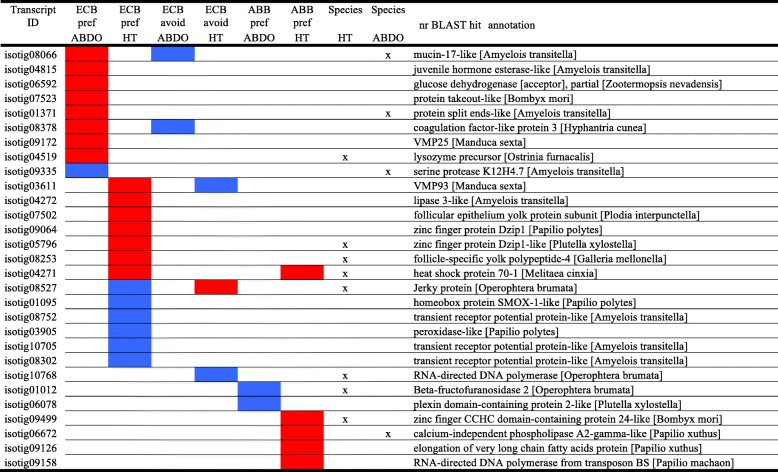
DE genes involved in ECB-pref, ECB-avoid and ABB-pref

For clarity, transcripts with no hits or with anonymous annotation (e.g. ‘uncharacterized protein’) were removed. In red: up-regulated; in blue: down-regulated. Crosses are for genes differentially regulated between species. A more detailed table is given in the Additional file 2: (Table S9)

Genes with a |Log2FC| > 2 are presented with their best “nr” hit annotation

We observed enrichment in various GO categories for the DE ECB-pref, ABB-pref and ECB-avoid genes involving the metabolism of carbohydrates, lipids, proteins, and amino acids, the detoxification mechanisms, immunity, and the chemosensory repertoire (Table 4). These functions were also observed in the gene lists specific to ECB-pref, ABB-pref, and ECB-avoid contrasts (Additional file 2: Table S10).

Lastly, we considered the DE gene lists in which the expression also differed between ECB and ABB. A reverse transcriptase, various enzymes involved in digestion and detoxification (e.g. mucin, Dzip1, Beta-fructofuranosidase, phospholipase, and serine protease), an immune protein (lysozyme precursor), a heat shock protein, and an egg yolk protein (Table 4) were included. All these DE genes were involved in ECB-pref and ECB-avoid, making them the best candidates for the divergence driven by specialization to maize.

### DE transcripts in candidate sensory genes

We compiled a set of genes known to be involved in sensory perception which had been previously described in ECB, ABB and related species of the *Ostrinia* genus (Additional file 2: Table S11). This gene set, hereafter called “Senso-ref,” was used as a gene reference independent of ABB-ref and ECB-ref in order to focus on sensory genes. Some 109 chemosensory genes of the 139 Senso-ref candidates were expressed in at least one library (Additional file 2: Table S12). As for the de novo transcriptome, most of the variation in expression for these genes was due to tissue type (ABDO vs. HT) with 65 DE genes, followed by moth species (ECB vs. ABB) with 36 DE genes. Overall, 28 genes were differentially expressed depending on the experimental conditions, of which a majority (24) differed between pure mugwort and pure maize conditions. When we focused on DE candidate genes potentially involved in traits and behaviors (ABB-pref, ECB-pref and ECB-avoid) observed at the phenotypic level (Additional file 1: Box S1–2, Additional file 2: Table S12) using specific contrast analysis in the GLM procedure, we highlighted eight genes involved in maize preference in ECB samples: four odorant receptors (ORs), one aldehyde oxidase, one carboxylesterase, and one ionotropic receptor (IR). Only two genes (ORs) were found in mugwort avoidance in ECB samples, and three genes (ORs) in mugwort preference in ABB samples. Notably, genes differentially expressed in ECB-pref differed from those detected in ABB-pref and ECB-avoid (Additional file 2: Table S9). Finally, of the 28 genes differentially expressed depending on the plant environment, 11 (5 ORs, 5 IRs and 1 carboxylesterase) were also differentially expressed between ABB and ECB samples.

## Discussion

Using an RNA-Seq approach without a priori and a specific analysis targeting chemosensory genes, we searched for genes expressed in ECB and ABB adult females exposed to various environments with or without their usual host plant. We identified genes and functions that differentiated ECB from ABB whatever, or depending on, the environment. We also classified these DE genes according to their expression pattern to relate them to preference and avoidance behaviors in ECB and ABB gravid females. In this way we identified candidate genes for species divergence by host specialization.

### Response to plant environment in ECB and ABB adult females: Global pattern

On average across all transcripts, we observed first of all that more genes were up- or down-regulated in HT tissues than in ABDO tissues. This observation corroborated the expectation that sensory and neuronal signals, which are mainly active in HT organs, would be major factors in oviposition [66, 80]. Second, we recorded more DE genes in ECB samples than in ABB samples. We may assume that this result is a signature of the strength and variability of host specialization in ECB (combining host preference and alternative host avoidance), while ABB appeared less specialized to its own host in terms of physiological and behavioral traits ([59], Additional file 1: Box S1). Lastly, we found that the genes involved in the preference for their hosts differed between ECB and ABB, as well as those involved in mugwort rejection in ECB, suggesting that the ECB shift onto maize was characterized by genetic novelties.

Response to plant environment in ECB and ABB adult females involved many processes, including metabolism of carbohydrates, lipids, proteins, and amino acids, detoxification mechanisms, immunity and chemosensory repertoire. These functions are key processes of host specialization in phytophagous insects [77], but were until now mainly illustrated at the larval stage [16, 29, 32, 43, 63, 76]. In the present study, we provide novel results for the adult stage, revealing some other candidates for host specialization like for example the Takeout-like proteins, previously reported as involved in circadian rhythms and feeding behavior in *Drosophila* [71].

### Chemosensory genes in host choice: New candidate genes and divergence between ECB and ABB

Targeted analyses of chemosensory genes supported their implication in host discrimination in ECB, ABB, or both. Moreover, five ORs, five IRs and one carboxylesterase appeared as suitable candidates for their putative role in species divergence by specialization. Indeed, their expression responded to plant environments and, at the same time, differed between moth species. Among these genes, one OR (LC002733.1, Additional file 2: Table S12) was particularly relevant, because it clearly discriminated between maize and mugwort conditions in ECB, either for maize preference or mugwort avoidance. In contrast to many other ORs involved in pheromone recognition by males, Wanner et al. [82] and Yang et al. [85] observed that this OR was expressed higher in ECB females. We suggest that the DE analysis may lead to the discovery of new potential candidates involved in plant odor discrimination in females during oviposition. Lastly, among the genes differentially expressed between moth species and potentially involved in ECB-pref, we identified one OR (JN169130, [40]) previously reported as more expressed in males than in females in ECB and the Asian corn borer (ACB), another sibling species of the *Ostrinia* species complex. This receptor may thus be an example of pleiotropy or synergetic activity for pheromone recognition in males and plant recognition in females. An essential requirement for speciation via specialization is the existence of a link between reproductive isolation and specialization due to divergent environment selection [51]. In ECB and ABB, two pheromone lineages coexist and are partially reproductively isolated due to the discrimination by males of the pheromone blends emitted by females [23, 37]. A gene implicated in both pheromone recognition by males and host plant discrimination for oviposition by females would be a direct link between mating isolation and host specificity [81].

### Major role of metabolism of organic compounds and divergence between ECB and ABB

Besides the recognition of plant chemical cues, the analysis based on whole de novo assembly highlighted a wide variety of other functional categories involved in either moth species divergence, host plant choice, or both.

The organic metabolic processes represented by key genes previously identified and involved in the differentiation between ECB and ABB [60] as well as in various phytophagous lepidopterans [77] highlighted the importance of the digestion and detoxification of plant metabolites, in particular at the larval stage. At the adult stage, chemosensory processes have been emphasized, because ovipositing females use plant secondary metabolites as cues to locate and select suitable plants [79]. Our study suggests that the digestion and detoxification of non-volatile compounds are also important factors in host choice. This result may be unexpected because ECB is often considered as a “capital breeder,” i.e. as an insect that feeds only during the larval stage, as is often the case for lepidopterans [7]. On the contrary, our results and previous work [13, 39] show that nutrition (and in particular carbohydrates) may play a key role in oviposition behavior, egg weight, fecundity and female longevity.

Interestingly, the activation of genes belonging to specific metabolic pathways differed between ECB and ABB, with for example mucin, serine protease and phospholipase genes involved in ECB-pref and ABB-pref. Hence these genes require further investigation to test whether they reflect differences in the metabolic strategies employed by ECB and ABB to survive, develop, and reproduce on their respective hosts.

### Unexpected involvement: Virus and retrovirus-like transposon

Besides the metabolism of organic compounds, in most DE genes we also observed functions relating to detoxification (cytochrome P450) and an immune-induced protein previously characterized in *Ostrinia* species (gb|AGV28583.1, Additional file 2: Table S6). These immune and detoxification genes may be mobilized to detoxify plant metabolites as well as being involved in the defense against bacteria and viruses. Indeed, we observed the genes of plants, viruses and bacteria, which were probably due to remnants of the food bolus or the presence of environmental, commensal, symbiotic, or pathogenic microorganisms. The RNA sequencing was not designed to target the microorganism community, so a fine and quantitative analysis would be unwarranted. Yet the presence of some of this alien fauna impacted the gene expression of ECB and ABB females, as illustrated by the activity of DNA-directed RNA polymerases (Additional file 2: Table S6) implicated in RNA virus replication. In particular for viruses, we observed contrasting patterns between ECB and ABB and between experimental conditions, with a higher prevalence of cypovirus-like genes in ABB HT samples in maize conditions than in ECB or other conditions (Additional file 2: Table S5-A). Thus we suggest that immune and defense genes differentially expressed between ECB and ABB, or between maize and mugwort conditions, may have been activated to contend pathogens present in the experimental environment. Interestingly, the prevalence of these microorganisms as well as the defense response of female moths differed between ECB and ABB samples, and between environments. These results, and previous similar observations at the larval stage [60], warrant further studies in other moth populations and plant species, to *i)* evaluate the variability and structuration of the microorganism communities in situ, *ii)* measure the immune response and its divergence between ECB and ABB, and *iii)* characterize the interactions between host plants, microorganisms and species divergence. Such multitrophic interactions have been demonstrated in different studies [64]. They merit further investigation to gauge their importance in the context of ECB–ABB divergence, since we cannot exclude opportunistic infections in experimental populations through chance or stress in a single treatment, rather than a generalizable biological reality.

Additionally, we observed the expression of retroviral-like transposons (Additional file 2: Table S5-B) and various genes associated with their transposition and spread, in particular in HT tissues of ECB. A putative role for transposable elements in ECB and ABB divergence has not yet been reported, but these elements are known to interfere with the adaptive response to various environmental stresses in various other taxa [11]. Bursts of transposable elements may thus in some circumstances be a driver of speciation events [5]. In ECB, Lillehoj [45] proposed a scenario in which aflatoxin (a toxin produced by the microbial inhabitants in the insect’s digestive tract) induced the activation of mobile genetic elements such as transposons, plasmids and chromosomal mutation, in response to environmental changes. According to this author, aflatoxin-producing fungi may be symbiotic in certain equilibrium conditions and pathogenic in conditions of nutritional imbalance, notably when elevated levels of digestible carbon compounds are present. The ecological disequilibrium caused by crop monoculture is supposed to have favored ECB populations with two or more life cycles per crop rotation over univoltine populations prone to a diapause period. Moreover, aflatoxin properties in host DNA and transposon activation may have facilitated this novelty in ECB. No similar mechanism has been described in ABB, and further studies are required to directly test the role of transposon activity in ECB-ABB divergence and maize colonization by ECB.

## Conclusions

Our results highlighted some potential candidate genes and functions involved in specialization and in species divergence between ECB and ABB. Through ad-hoc categorization, we discriminated between genes in ECB and ABB that responded to the environment with similar or divergent patterns. Independent approaches such as functional and population genomics will be required to confirm their role in the history of ECB host shift onto maize. Besides, we confirmed the role of sensing processes and identified new candidates for the chemosensory repertoire. More precise studies must now be conducted to characterize the role of these candidates in specific traits of the female adult stage such as oviposition, mating, or metabolism, since gene expression is potentially highly sensitive to many environmental factors. These candidates should also be tested in different populations and contexts, since they could help with the design of effective control solutions for crop pest management [65]. Finally, we highlighted other avenues for research on carbohydrate metabolism and virus and retrovirus dynamics, which hold the promise of enhancing our understanding of divergence between cryptic sibling species.

## Methods

### Moth sampling and rearing

In this study, we sequenced RNA from adult moths sampled from a previous experiment [59], where phenotypic and behavioral traits in moths faced with different plant environments were measured. The moths in the experiment originated from a single generation of laboratory-reared larvae initially sampled in maize and mugwort stands near Versailles (northern France). In this region, several genetic studies had verified that the host plant (maize vs. mugwort) was the best criterion for discriminating between ECB and ABB samples [47, 48, 52]. Hence we then applied the nomenclature proposed by Frolov et al. [19] for specimens collected on maize and mugwort respectively.

The experimental framework is summarized in Additional file 1: Box S1 under “Experimental design”. Briefly, around 20 males and females were released into three types of conditions: (*i)* “choice” cages, with maize and mugwort plants, (*ii)* “maize” cages (the usual host for ECB) and (*iii)* “mugwort” cages (the usual host for ABB). After 72 h, several life history traits, and particularly egg laying, were quantified. After the measurements, all adults were recaptured and the females were used for the acquisition of expression data by RNA sequencing (see figure in Additional file 1: Box S1).

At the time of sampling, we expected most females to be searching for favorable oviposition sites, because in the female adult lifetime (ca. 10–20 days in the laboratory) most females begin to mate and lay eggs 2 days after emergence ([24, 58] and pers. obs.). They continue to lay eggs for several days (with or without additional mating) with egg laying activity peaking at about 10 days. Sampling after 72 h was thus the best compromise between sufficient egg laying and low mortality. This sampling method aimed at capturing the gene activity underlying oviposition behavior and physiology (host searching, egg maturation and laying) by maximizing the number of females ready to oviposit, although some females in the sample were probably not in that target state.

### Dissection, RNA extraction and sequencing

As we primarily targeted genes involved in host preference and host avoidance during oviposition, we focused on females only. Moreover, we assumed that these traits certainly involved chemosensory genes. Chemical reception is mediated by specialized sensory neurons located in the antennae, mouthparts or legs, but chemosensory genes are also involved in non-sensory functions and expressed in other tissues [84]. Thus, we chose to split by tissue samples into head-thorax (HT) and abdomen (ABDO) tissues. HT tissues contained the legs, antennae, and mouthparts while ABDO tissues contained the digestive and genital organs. In addition, the use of separate pools increased the sequencing coverage per pool of tissue. In total, we dissected 220 females on ice to prevent thawing and RNA degradation. Total RNA was extracted from each tissue and individual with the AllPrep DNA/RNA 96 Kit (Qiagen), according to the manufacturer’s protocol. RNA quality and quantity were assessed using a NanoDrop (ThermoFisher), and equimolar quantities of individual extracts were pooled to obtain 24 samples corresponding to 2 repetitions × 3 experimental conditions × 2 tissues × 2 moth species. Each pool contained 7 to 11 females depending on the RNA samples available (Additional file 1: Table S4).

Twenty-four cDNA libraries were constructed with the TruSeq Stranded mRNA Sample Preparation Kit (Illumina) according to the manufacturer’s protocol and sent to the MGX platform (Montpellier, France) for single-end 1x50bp sequencing on six lanes of a HiSeq 2000 (Illumina).

### De novo transcriptome assembly and annotation

After sequencing, all reads were subjected to quality control and trimming using Trimmomatic (version 0.25) to remove Illumina sequencing adapters and low quality reads [6]. Ribosomal and bacterial contaminants were identified using sortmeRNA ([33] version 2.0, default parameters), and Bowtie2 ([35]; version 2.2.4) mapping against a bacteria subset dataset extracted from the NCBI database (October 2014).

First, we applied default parameters and the normalization procedure of the Trinity package (version r20140413p1 [28]) to assemble high quality reads of the 12 ECB libraries and of the 12 ABB libraries separately into two comprehensive transcriptomes. Then, for each of the resulting ECB and ABB assemblies, we included a 454 de novo transcript set previously obtained by Gschloessl et al. [27] from four ECB and four ABB adults, respectively. Such meta-assemblies that combine different technologies of sequencing reads have been proved to increase contig length and coverage in other studies [50, 61]. To achieve this goal, we used the EMBOSS [68] splitter program (version 6.6.0) to create short overlapping subsequences (length 300 bp, overlap 200 bp) for each of the 454 transcripts and HiSeq transcript sets, respectively. All 454 and HiSeq subsequences were then assembled with Newbler (454 Life Sciences Corporation, version 2.9) set at 40 bp for minimum overlap length, 97% for minimum overlap identity, and ‘Het’ (for heterozygote). Finally, we selected the longest transcript as the representative sequence for each gene to circumvent any putative alternative transcripts that might bias the expression analyses.

For functional and GO-term annotations, a custom script was developed to compare ECB-ref and ABB-ref genes against NCBI nr (version May 2016) and UniProtKb ([4], version June 2016) using *blastx* BLAST (blast+, version 2.2.28, [9]) and InterProScan [31].

### Candidate sensory genes

In addition to the reconstruction of the de novo transcripts, we compiled a set of 141 reference genes involved in sensory perception previously described in ECB, ABB and related species of the *Ostrinia* genus [40, 53, 54, 82, 85, 86]. They included odorant receptors (ORs), gustatory receptors (GRs), ionotropic receptors (IRs), sensory neuron membrane proteins, and odorant degrading enzymes. As some of them may be orthologous between species, or similar between studies, we removed the redundant sequences as identified by reciprocal BLAST analyses. After this filtering step, the sensory dataset was reduced to 139 unique sequences (Additional file 2: Table S11). This gene set, “Senso-ref”, was used as an independent gene reference from ABB-ref and ECB-ref to focus on sensory genes.

### Raw read mapping and normalization

Raw reads were mapped onto ABB-ref, ECB-ref and Senso-ref using Bowtie2 ([35], version 2.2.4), set to the default parameters and “sensitive.” After mapping, we produced mapped read counts for each gene using the SAMtools program [44]. Transcript-specific variations (the dispersion and logarithmic fold-changes of read counts across the experimental conditions) were estimated with the Bioconductor EdgeR (Empirical Analysis of Digital Gene Expression Data in R) package ([69], version 3.8.6). A negative binomial distribution was chosen as the model for count variations, and transcripts with less than one count per million for at least half of the libraries were removed. Read counts were normalized across the conditions (i.e. across the sequenced libraries) using the *calcNormFactors* EdgeR function and TMM option [70]. Log_2_ fold changes (Log2FC) were calculated for each gene between conditions.

In addition, a principal component analysis (PCA) was conducted on the normalized read counts, as implemented in the FactoMineR library of R [38]. The following categorical variables were inputted as supplementary variables: the moth species (ABB vs. ECB), the experimental condition (choice, mugwort, maize), and the tissue (ABDO vs. HT).

### Identification of differentially expressed genes: response to environment and divergence between ECB and ABB

The main source of variation in expression was due, as expected, to the HT vs. ABDO tissues rather than to the species or experimental conditions (Fig. 1). Thus, we analyzed the ABDO and HT libraries separately. Variations in read counts by transcript (*t*_*i*_) were analyzed using the EdgeR linear model to estimate the statistical evidence for the experimental effects. We combined the two explanatory variables *moth species* and *experimental condition* into a so-called *“modalities”* factor, and applied the following model:

Model 1: ti = modalities.

We then used a contrast analysis to identify *i)* transcripts whose expression varied significantly between ABB and ECB whatever the experimental condition, and *ii)* transcripts whose expression varied significantly between the experimental conditions (maize and mugwort, maize and choice, mugwort and choice) whatever the moth species. Transcripts were considered differentially expressed (DE) across the entire experiment if they had a false discovery rate (FDR) adjusted *p-value* < 0.01.

Gene ontology (GO) enrichment was calculated in R using topGO package (version 2.26.0) from the Bioconductor project [1]. We used the GO annotation described previously as a custom input. We removed GO terms with fewer than five annotated genes (using the *nodeSize* parameter set to 5). We used the weight algorithm and Fisher’s exact test. We reported enriched GO categories with *p-values* < 0.01 for DE genes between ECB and ABB species, and for DE genes between the experimental environments (maize and mugwort, maize and choice, mugwort and choice).

### Categorization of DE genes according to behavioral traits

We further refined the categorization of DE genes to relate them to the preference and avoidance traits and behaviors previously observed in Orsucci et al. [59] (Additional file 1: Box S1). To do this, we split the data set and analyzed the ECB and ABB data separately using a simplified model:

Model 2: ti = experimental condition

We compared *i)* gene expression between mugwort samples and the mean expression of the two other experimental conditions in the ECB sub-data to detect genes potentially involved in maize preference during oviposition in ECB (hereafter referred to as ECB-pref); *ii)* gene expression between maize samples and the mean expression of the two other experimental conditions in the ABB sub-data to detect genes involved in mugwort preference during oviposition in ABB (ABB-pref); and *iii)* gene expression between maize-only and the mean expression of the two other experimental conditions in the ECB sub-data to detect genes involved in mugwort avoidance during oviposition in the mugwort environment in ECB (ECB-avoid).

For all models, the *p-values* were adjusted for multiple testing using the Benjamini-Hochberg procedure (FDR < 0.01). GO enrichment was calculated as previously for the ECB-pref, ABB-pref and ECB-avoid gene lists.

## Additional files

Additional file 1: **Box S1.** Experimental framework of RNAseq sequencing: material, methods and main results on the behavior and life history traits of ECB and ABB during the oviposition phase (derived from [59]). (PDF 574 kb)

Additional file 2: **Table S1.** Details on sequencing reads and contaminants per library. **Table S2.** Top hit species after BLAST analysis, for the ECB-ref transcripts (sheet 1) and ABB-ref transcripts (sheet 2). **Table S3.** Read mapping against ABB-ref transcripts for ABB and ECB samples. **Table S4.** Sequencing characteristics of the RNA pools. **Table S5**. Detailed information for DE genes homologous with virus (A) and retrovirus-like transposon sequences (B). **Table S6.** Detailed information for genes DE between ECB and ABB species. **Table S7.** Fisher’s test of enrichment in GO term categories between the DE transcripts (test set) and the whole transcriptome (reference set). **Table S8**. Detailed information for genes DE between experimental environments (maize, choice, mugwort). **Table S9.** Detailed information for genes involved in ECB-pref, ECB-avoid and ABB-pref. **Table S10.** Detailed information for genes specific to ABB-pref, ECB-pref and ECB-avoid contrasts in HT and ABDO tissues. **Table S11.** Published chemosensory *Ostrinia* spp. genes. **Table S12.** Detailed information for DE chemosensory genes.

Additional file 3: **Figure S1.** Venn diagram for ECB-ref (A and B) and ABB-ref (C and D) transcripts shared between the different experimental conditions for the HT samples (A and C) and ABDO samples (B and D). (PDF 183 kb)

## Abbreviations

ABB: Adzuki bean borer
ABDO: Abdomen
avoid: Avoidance
bp: Base pair
DE: Differentially expressed
ECB: European corn borer
FC: Fold change
FDR: False discovery rate
GLM: Generalized linear model
GO: Gene ontology
GR: Gustatory receptor
HT: Head-thorax
IR: Ionotropic receptor
L4: Fourth larval instar
LRT: Likelihood ratio test
Mbp: Mega base pair
OR: Odorant receptor
PCA: Principal component analysis
pref: Preference
TMM: Trimmed mean M values
