# Supplementary material for: Transcriptomic response of female adult moths to host and non-host plants in two closely related species
Source: BMC Evol Biol. 2018 Sep 20;18:145. doi: 10.1186/s12862-018-1257-3 (PMC6148789; doi:10.1186/s12862-018-1257-3)
Supplement: Supplementary file 1 — Box S1. Experimental framework of RNAseq sequencing: material, methods and main results on the behavior and life history traits of ECB and ABB during the oviposition phase (derived from [59]). (PDF 574 kb) [file 12862_2018_1257_MOESM1_ESM.pdf]

Box S1 : Experimental framework of RNAseq sequencing : material, methods and main results on the behavior and life history traits of ECB and ABB during the oviposition phase (derived from Orsucci *et al.* 2016)

1. *Experimental design*

ECB and ABB larvae were collected near Versailles, France (48°48019″N, 2°08006″E) from maize and mugwort stands, respectively (Fig B1.A). Mating and growth of the next generations were conducted in laboratory conditions according to Orsucci *et al.* (2016). After the fifth instar molting, each F2 pupa was isolated until emergence to ensure adult virginity before use in experiments (Fig B1.B). Some 20 females and 15 males were released into three different experimental conditions: "maize" (*i.e.* pure maize), "mugwort" (*i.e.* pure mugwort), and "choice" (*i.e.* mixture of mugwort and maize) for the oviposition experiment. After 3 nights (~72h) in semi-natural conditions, behavioral and phenotypic traits were measured. The recovered adults were flash-frozen in liquid nitrogen and stored at -80°C (Fig B1.C). Finally, each frozen female was cut in two to separate head and thorax tissues from abdomen tissues.

One equimolar pool of RNA extracts was taken per cage (Fig B1.D). A total of 24 RNA libraries corresponding to 2 moth species x 3 experimental conditions x 2 repetitions were sequenced in a 1x50bp design on a HiSeq2000 (Illumina).

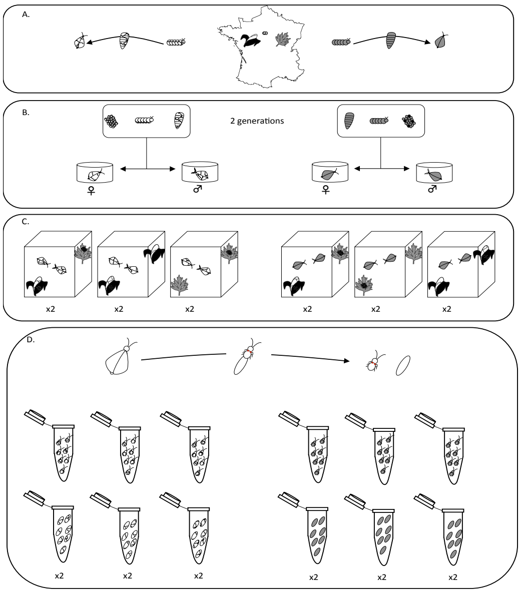

Experimental design from sampling in natural populations to RNA extractions. ECB and ABB are represented in white and gray, respectively.

2. *Summary of variations in life history traits in ECB and ABB females during oviposition*

We measured variations of life history traits in ECB and ABB gravid females in the three experimental conditions: "maize", "mugwort", and "choice":

(i) fertility was estimated as the total number of eggs laid in cages;

(ii) host preference for oviposition was significant when ECB laid more eggs on maize than on mugwort plants in choice conditions, and *vice-versa* for ABB;

(iii) host avoidance for oviposition was significant when ECB (or ABB) females laid more eggs on a neutral site (cage netting) than on plants when they had no choice but mugwort (maize) available;

(iv) survival was measured as the number of moths recovered after 3 days of release in the experimental cages compared to the number of moths initially released;

(v) resting site concerned the site of capture: mugwort, maize or cage netting.

The results observed for these traits are summarized in the table below.

Trait	ECB		ABB	
	Maize	Mugwort	Maize	Mugwort
Fertility	++	--	-	+
Host *preference* for oviposition	+++	---	-	+
Host *avoidance* for oviposition	NO	YES	NO	
Survival	+	-	=	
Resting	+	-	=	

Main results of oviposition experiment: +/- signs (and YES/NO) indicate the direction and strength of significant relationships between traits, moth species and experimental conditions. The '=' sign indicates no effect. (Derived from Orsucci *et al.* 2016).
