# Supplementary figures and images for: Transcriptomic response of female adult moths to host and non-host plants in two closely related species
Source: BMC Evol Biol. 2018 Sep 20;18:145. doi: 10.1186/s12862-018-1257-3 (PMC6148789; doi:10.1186/s12862-018-1257-3)

## ECB-ref

A.

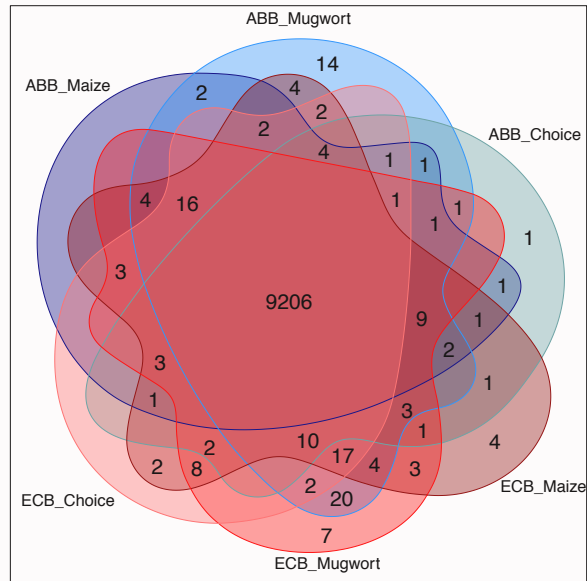

B.

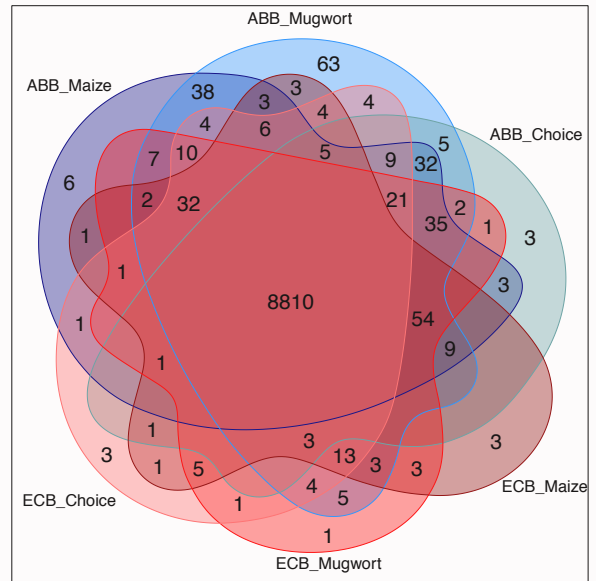

## ABB-ref

C.

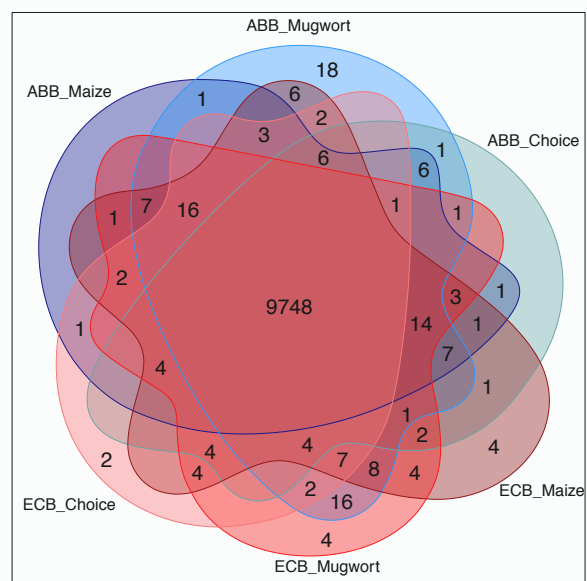

D.

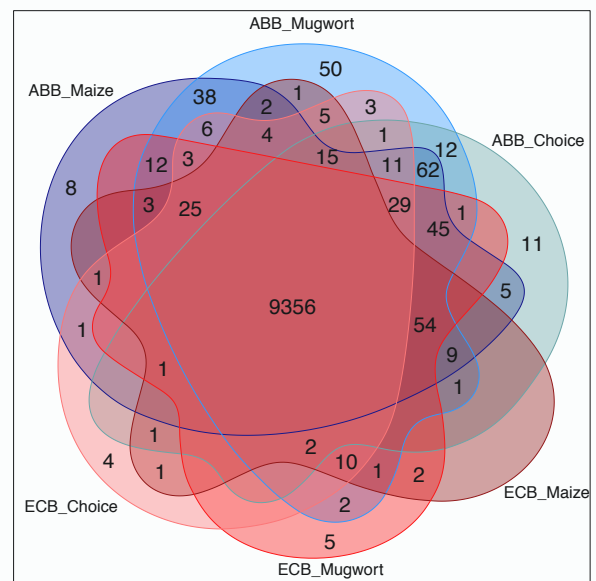

**Figure S1**

Supplement: Supplementary file 3 — Figure S1. Venn diagram for ECB-ref (A and B) and ABB-ref (C and D) transcripts shared between the different experimental conditions for the HT samples (A and C) and ABDO samples (B and D). (PDF 183 kb) [file 12862_2018_1257_MOESM3_ESM.pdf]
